# Portal flow modulation in living donor liver transplantation: review with a focus on splenectomy

**DOI:** 10.1007/s00595-019-01881-y

**Published:** 2019-09-25

**Authors:** Tomoharu Yoshizumi, Masaki Mori

**Affiliations:** Department of Surgery and Science, Graduate School of Medical Sciences, Kyushu University, 3-1-1 Maidashi, Higashi-ku, Fukuoka, 812-8582 Japan

**Keywords:** Living donor liver transplantation, Portal flow, Modulation

## Abstract

Small-for-size graft (SFSG) syndrome after living donor liver transplantation (LDLT) is the dysfunction of a small graft, characterized by coagulopathy, cholestasis, ascites, and encephalopathy. It is a serious complication of LDLT and usually triggered by excessive portal flow transmitted to the allograft in the postperfusion setting, resulting in sinusoidal congestion and hemorrhage. Portal overflow injures the liver directly through nutrient excess, endothelial activation, and sinusoidal shear stress, and indirectly through arterial vasoconstriction. These conditions may be attenuated with portal flow modulation. Attempts have been made to control excessive portal flow to the SFSG, including simultaneous splenectomy, splenic artery ligation, hemi-portocaval shunt, and pharmacological manipulation, with positive outcomes. Currently, a donor liver is considered a SFSG when the graft-to-recipient weight ratio is less than 0.8 or the ratio of the graft volume to the standard liver volume is less than 40%. A strategy for transplanting SFSG safely into recipients and avoiding extensive surgery in the living donor could effectively address the donor shortage. We review the literature and assess our current knowledge of and strategies for portal flow modulation in LDLT.

## Introduction

Due to severe organ shortages and the increasing gap between supply and demand, living donor liver transplantation (LDLT) has become an accepted alternative to expand the donor pool [1-4]. Variables such as the model for the end-stage liver disease score, donor/recipient age, recipient body mass index, and pretransplant diagnosis reportedly allow for the prediction of short-term mortality after liver transplantation [5–7]. However, the use of partial hepatic grafts in LDLT can complicate the prediction [8]. Since the introduction of adult LDLT, graft size has become a concern, particularly for patients with Child class C cirrhosis and/or portal hypertension. Small-for-size graft (SFSG) syndrome after LDLT is a major complication of this procedure [9-12]. It is defined as the dysfunction of a small graft within the first 1–2 post-transplant weeks in the absence of any other identifiable cause and is characterized by coagulopathy, cholestasis, ascites, and encephalopathy [13, 14]. A key mechanism of SFSG syndrome is thought to be excessive portal flow transmitted to the allograft in the postperfusion setting, resulting in sinusoidal congestion and hemorrhage [13]. In the setting of portal overflow, adaptive responses in the liver lead to vasoconstriction of the hepatic artery [15]. Whereas portal overflow injures the liver directly through nutrient excess, endothelial activation, and sinusoidal shear stress, arterial vasoconstriction introduces secondary ischemic damage [16, 17]. These insults may be attenuated with portal flow modulation. Attenuating portal flow increases arterial flow and improves graft function [18, 19].

Currently, a donor liver is considered a SFSG when the graft-to-recipient weight ratio (GRWR) is less than 0.8% or the ratio of the graft volume (GV) to the standard liver volume (SLV) is less than 40% [18, 20]. Factors other than the GV can potentially influence outcomes. These factors include recipient-related factors such as clinical disease status and portal hypertension; graft-related factors such as donor age, steatosis, and immunological factors, and technical factors such as vascular reconstruction, adequate hepatic venous outflow, and vascular inflow [8, 20–23]. For example, grafts within a wide range of GV-to-SLV ratios can tolerate portal hypertension if they have excellent venous outflow capacity, but when the ratio range is exceeded, modulation of vascular inflow becomes necessary for graft survival [19].

One Korean group no longer measures portal pressures routinely. Rather, they consider establishing excellent venous outflow, preventing acute rejection, and having young donors to be key mechanisms for avoiding SFSG syndrome in grafts with a GRWR as small as 0.7%. That group noticed that successful SFSGs were from young donors with young recipients in whom perfect venous outflow was ensured [24]. They maintain that portal venous pressure (PVP) > 20 mmHg is not associated with an increased risk of SFSG syndrome or graft loss, as long as perfect venous outflow is maintained and portal flow steal is interrupted with portosystemic shunt ligation [13, 24].

It is important to consider donor safety and avoid subjecting healthy living donors to excessive surgery [25–28]. A strategy for transplanting SFSG safely into recipients while avoiding excessive surgery in living donors could effectively address the donor shortage [20]. Several strategies to prevent SFSG syndrome, including portal flow and/or hepatic venous outflow modulation, have been reported [4, 9, 29]. Excessive portal flow and/or the reduced intrahepatic vascular bed result in higher portal flow, increased portal pressure, and stress at the hepatic sinusoid [30, 31]. To reduce the risk of these factors, different portal flow modulation techniques have been described. We review the relevant literature and assess the current knowledge of and strategies for portal flow modulation in LDLT.

## Splenectomy

Generally, splenectomy is performed to reduce the bleeding tendency resulting from thrombocytopenia, or as part of surgical procedures such as devascularization of the upper stomach and esophageal transection to control variceal hemorrhage. It is also performed for hematologic disease such as idiopathic thrombocytopenic purpura, splenic tumors such as malignant lymphoma, trauma, splenic artery aneurysm, and liver or renal transplantation from an ABO-incompatible donor [32]. In rodent models, splenectomy improves the vascular compliance of the graft and increases hepatic serotonin, which plays a significant role in hepatic perfusion through its vasodilatory effects [33, 34]. Hepatic serotonin improves microcirculation and promotes liver regeneration by stimulating the endothelial cells to release vascular endothelial growth factor. It also protects the graft by increasing the microcirculation and accelerating liver regeneration [35].

Simultaneous splenectomy during LDLT improves graft outcomes by reducing the portal pressure and flow and increasing the vascular compliance of the graft [36–38]. We previously reported that simultaneous splenectomy reduced hypersplenism and prevented graft congestion resulting from excessive portal flow [9, 39]. In our first report, the outcomes of six cases of LDLT with a left-lobe graft were analyzed. None of the patients who underwent splenectomy suffered hyperbilirubinemia or intractable ascites. Both portal pressure and portal vein flow decreased after splenectomy in most of the patients. To clarify whether splenectomy was beneficial for patients with a SFSG, further analysis was performed for patients who had a GV-to-SLV ratio of 40% or lower (*n* = 50) [9]. SFSG syndrome developed in 11 of 50 patients with a GV-to-SLV ratio of 40% or lower, and excluding splenectomy was an independent risk factor for SFSG syndrome in our patients. Kaido et al. clarified that overall survival rates after LDLT were significantly higher for patients with a final PVP ≤ 15 mmHg than for those with a PVP > 15 mmHg [40]. Therefore, they routinely apply a portal pressure control program that targets a final PVP ≤ 15 mmHg to prevent SFSG syndrome. The Kyoto group recently reported re-evaluating the indications for PVP modulation, which they achieve primarily with splenectomy [41]. They found that failed PVP modulation (final PVP > 15 mmHg) was associated with an increased incidence of SFSG syndrome and early graft loss. Among 38 patients with failed PVP modulation, donor age ≥ 45 years and ABO incompatibility were independent risk factors for graft loss [41]. Survival analysis showed that PVP > 15 mmHg was related to poor prognosis in grafts from either ABO-incompatible donors or from donors ≥ 45 years of age, but it did not negatively affect grafts from ABO-compatible/identical donors or from donors < 45 years of age. They concluded that PVP modulation is not necessary in all recipients. Grafts from ABO-compatible/identical donors and from donors < 45 years of age can tolerate portal hypertension; however, lowering the final PVP to ≤ 15 mmHg is necessary for patients with grafts from ABO-incompatible donors or from donors ≥ 45 years of age [41]. In most reports, the decision to perform splenectomy was made after graft implantation. Portal hyperperfusion can injure the SFSG when splenic flow is present. Therefore, we perform splenectomy before graft implantation to prevent graft injury if portal hypertension is anticipated, because of splenomegaly and excessive portal flow. Among 258 patients who underwent splenectomy for portal flow modulation during LDLT at Kyushu University, 232 (89.9%) underwent splenectomy before implantation. In a rodent SFSG liver transplant model, better outcomes were achieved when splenectomy was performed just before partial liver transplantation (LT) than after partial LT because of the direct elimination of splenic inflammatory leukocytes and inhibition of inflammatory leukocyte infiltration [42].

In Japan, the benefits of simultaneous splenectomy in LDLT are well documented, whereas the negative effects, including potential adverse events such as portal venous thrombosis (PVT), infectious complications, pancreatic fistula, and postoperative bleeding, are not discussed in detail [43]. Ito et al. recently reported a significantly higher incidence of reoperation for postoperative hemorrhage within the first postoperative week, and of lethal infectious disease as well as greater intraoperative blood loss and longer surgery time among recipients who undergo simultaneous splenectomy than among those who do not [43]. Selected patients in that series underwent splenectomy at the time of LDLT if medically indicated, but never for PVP modulation. Patients who underwent splenectomy did not have a lower incidence of SFSG syndrome than those who did not. Notably, a GV-to-SLV ratio above 40% was required and the donors [43] were younger than those in the Kyoto study (median, 35 years old vs. 45 years old, respectively) [41]. A vessel sealing system for dissection around the spleen and a vascular stapler for the splenic hilum have improved the ease and safety of splenectomy, even for patients with severe portal hypertension [9, 44]. Furthermore, the splenic artery is routinely ligated when simultaneous splenectomy is performed. Consequently, intraoperative blood loss and surgery time are not increased when simultaneous splenectomy is performed at our center. However, more technical refinement is necessary to prevent pancreatic fistula and bleeding from the splenic stump.

## Portal vein thrombosis after splenectomy

PVT is a severe complication of LDLT that can result in increased morbidity and mortality [45]. The incidence of PVT in deceased donor LT ranges from 0.3 to 2.6% [46, 47], and increases to 4–9% in adult LDLT with more complex surgical techniques and complicated vascular reconstructions, mainly related to shorter vessel grafts resulting in shorter vessel length for anastomosis [48, 49]. PVT is not a rare complication of splenectomy for patients with cirrhosis in a non-transplant setting [32, 50]. In patients with cirrhosis, decreased portal flow and the development of portosystemic collaterals are considered predisposing factors for PVT. Furthermore, the imbalance between coagulation factors and coagulation inhibitory factors resulting from decreased levels of coagulation inhibitory factors such as protein C, protein S, and antithrombin-III, may cause PVT in these patients [32, 50]. Kinjo et al. also reported that the incidence of PVT after splenectomy in a non-transplant setting was 24.3%. The independent risk factors for PVT in that study were large splenic vein diameter (13 mm or more) and low white cell count (≤ 2 × 10^3^ /mm^3^), and spleen weight was correlated with splenic vein diameter and white cell count [51].

**Fig. 1:**
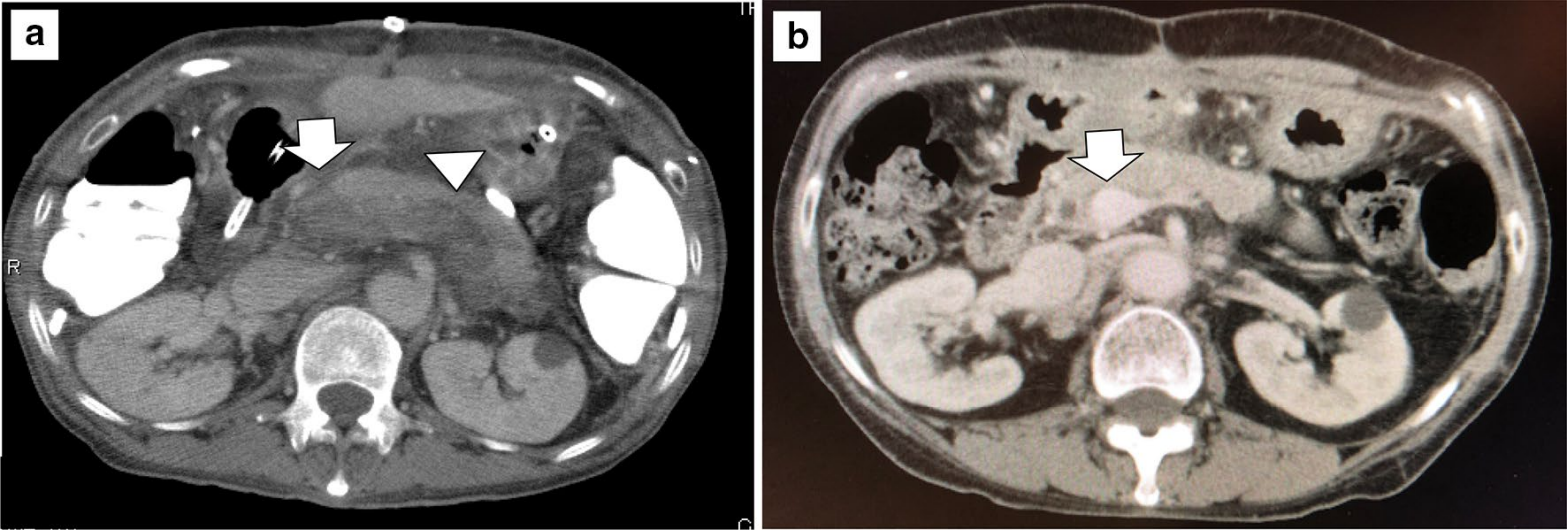
Representative case of portal vein thrombosis (PVT). **a** The PVT (arrow) extended from the splenic vein (arrowhead) stump 3 days after living donor liver transplantation (LDLT) with simultaneous splenectomy. **b** Enhanced computed tomography (CT) shows a patent portal vein (arrow) 1 year after LDLT

**Fig. 2:**
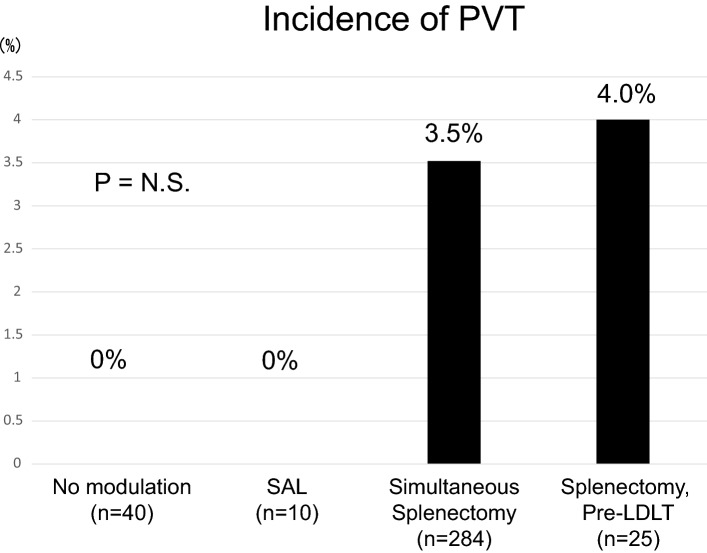
Incidence of portal vein thrombosis (PVT). The incidence of PVT after LDLT was 3.5% with simultaneous splenectomy and 4.0% with splenectomy before LDLT. In contrast, no PVT was detected in patients without portal flow modulation or in those who underwent SAL. The difference among the groups was not significant

The relationship between splenectomy and an increased incidence of PVT after LDLT is not clear. Kurata et al. reported a very high incidence of PVT (33.3%) after LDLT with splenectomy, but no incidence after LDLT without splenectomy [52]. They concluded that using grafts of sufficient size was the key to controlling PVP and that splenectomy, a risk factor for PVT, should be avoided whenever possible in LDLT [52]. Blood stasis in the stump of the splenic vein results in thrombosis, which subsequently extends to the portal and superior mesenteric veins [32, 51, 53]. Figure 1 shows a PVT extending from the splenic vein stump after LDLT with simultaneous splenectomy. This patient underwent emergency thrombectomy, which was effective and there was no recurrence of PVT. Anticoagulant therapy was not given to this patient. The Kyoto group reported that the incidence of PVT did not differ between patients with splenectomy (5.7%) and without (2.6%), whereas the incidence of isolated splenic vein thrombosis was higher in patients with splenectomy (6.8%) and required short-term anticoagulant therapy [53]. Although patients with isolated splenic vein thrombosis are not given anticoagulants at Kyushu University, the incidence of PVT after LDLT is not higher at this institution than in the Kyoto group (Fig. 2). Further study is needed to establish whether anticoagulant therapy is recommended for patients with splenic vein thrombosis after splenectomy.

## Splenic artery ligation

Splenic artery ligation (SAL) was initially applied to prevent thrombocytopenia in LDLT [54]. Troisi et al. proposed the use of SAL to resolve ascites and increase hepatic arterial flow in LDLT [55]. Moreover, they reported that SAL was a simple and effective method for decreasing portal flow when recipient portal venous flow did not exceed 500 ml/min per 100 g of liver. Other techniques, such as portocaval shunt or portomesenteric disconnection should be considered when SAL is insufficient to relieve portal hypertension [56]. Therefore, SAL is an alternative to splenectomy for reducing PVP and flow [57]. Ishizaki et al. reported their successful experience of LDLT using left-lobe grafts without portal flow modulation [58]. In their study, the mean GRWR was 0.82% and 6 of the 42 patients underwent SAL. At Kyushu University Hospital, recipients who underwent SAL had worse graft function after LDLT than those who underwent splenectomy [9, 39]. Moreover, the 1-year graft survival rate was 91.2% for recipients with splenectomy, but only 77.9% for those with SAL in our single-center experience. This suggests that SAL was insufficient to modulate excessive portal flow and/or pressure compared with splenectomy. Umeda et al. established the preoperative proximal splenic artery embolization technique to prevent intraoperative bleeding resulting from injury to the massive collateral vessels around the splenic artery [59]. They embolized the proximal splenic artery with interventional radiology 12–18 h before LDLT without complications such as sepsis, portal thrombus, or abscess formation. They concluded that splenic artery embolization reduced excessive portal flow and improved graft function, which lowered the incidence of SFSG syndrome and had advantages for liver regeneration [60]. Since patients who wait for LDLT often have thrombocytopenia and coagulopathy, pseudoaneurysm of the punctured artery or bleeding around the puncture site should be considered and checked when performing this technique. Furthermore, this technique has a substantial risk of pancreatitis and postembolization syndrome [61]. Moon et al. recently reported the effects of a splenic devascularization procedure to prevent various complications, including PVT, pancreatic fistula, and bleeding from the splenic stump after splenectomy [61]. They performed not only SAL but also ligation of the right gastroepiploic artery and division of the gastrosplenic ligament including the short gastric arteries as an alternative to splenectomy. Since arterial supply to the spleen is maintained by intrapancreatic collaterals from the superior mesenteric artery, there was no incidence of splenic infarction and/or abscess. The splenic volume decreased to 60% of the original volume for 1 month after LDLT with splenic devascularization. Although the incidence of SFSG syndrome was similar in the two groups, procedure-related complications were less common in patients who underwent devascularization than in those who underwent splenectomy. The mean GRWR at their institution was 1.1% and splenectomy or devascularization was performed in only 10.6% of patients. Further studies from other institutions that use smaller grafts would clarify the impact of this procedure.

## Portocaval shunt

**Fig. 3:**
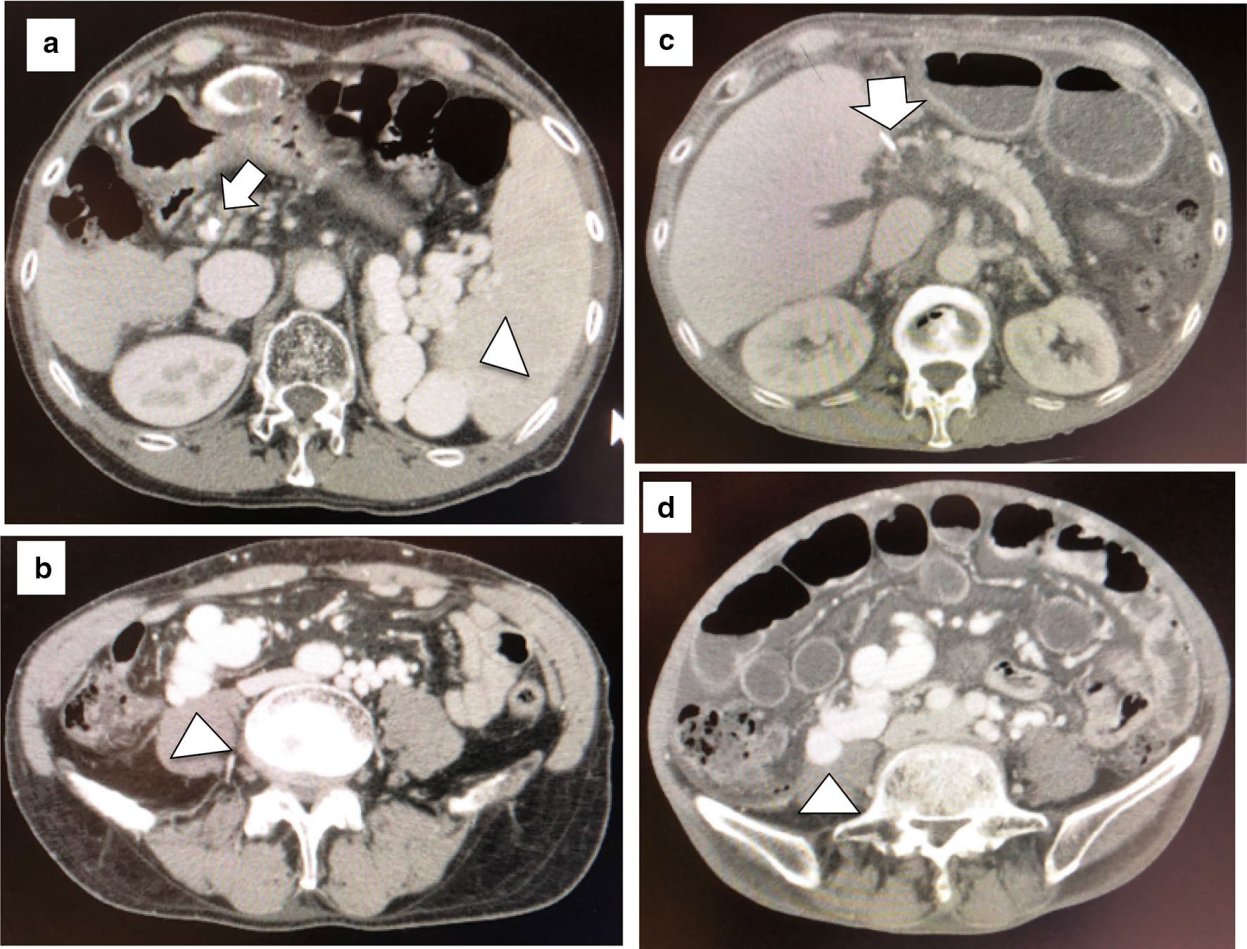
Representative case of a patient with a huge portosystemic shunt. **a**, **b** Pre-LDLT: enhanced CT shows huge splenorenal (arrow head) and mesocaval (arrowhead) shunts. The portal vein was atrophic (arrow). **c**, **d** 3 months after LDLT with splenectomy. Enhanced CT reveals a patent mesocaval shunt (arrowhead). The portal vein (arrow) was thrombosed. The splenorenal shunt was closed with simultaneous splenectomy

Compensatory portosystemic shunts develop in about 40% of patients with cirrhosis and the frequency increases with severity [62, 63]. Large shunts can be an advantage during recipient hepatectomy because the effect of severe portal hypertension is significantly reduced and portal clamping results in less congestion in the mesenteric system [63, 64]. Spontaneous portosystemic shunts are commonly removed during standard LT because the escape of portal inflow through collaterals (steal phenomenon) may lead to ischemic graft damage [65] and PVT (Fig. 3). Troisi et al. recommended that spontaneous portosystemic shunts be left in place when small grafts (GRWR < 0.8%) with hyperkinetic portal flow are used in LDLT [56]. The Kyoto group reported that the ligation of large portosystemic shunts increases portal venous flow and to prevent the portal venous steal phenomenon after LDLT when graft resistance increases during rejection [40]. The Asan group reported surgical interruption of portosystemic collaterals and additional evaluation of collaterals with intraoperative cine-portogram in recipients with large portosystemic shunts, which are a possible route of postoperative portal flow steal [61, 66, 67]. We ligate large shunts to maintain adequate portal flow and to prevent the steal phenomenon as long as the portal pressure does not exceed 20 mmHg after test clamping [8, 21, 68]. Balloon-occluded retrograde transvenous obliteration is a feasible intervention to close the remnant shunt when portal flow decreases because of the steal phenomenon [69]. Takatsuki et al. opposed mandatory ligation of shunts because 75% of their patients experienced no complications without ligation and the shunt ligation procedure can be dangerous [70].

To prevent graft failure resulting from portal hyperperfusion, diversion of the superior mesenteric flow with a mesocaval shunt was first described for LDLT using a SFSG [71]. Animal experiments using a portocaval shunt in SFSG transplant have demonstrated that adequate decompression of the portal system can effectively prevent the sinusoidal congestion and graft injury typically seen in SFSG syndrome [72, 73]. A hemi-portocaval shunt reduced portal flow and improved patient and graft survival by preventing SFSG syndrome in the clinical setting [74-76]. It is important to rule out hepatic venous outflow obstruction before creating a shunt because a shunt in the presence of this obstruction can cause severe portal steal and graft ischemia [63]. Hemi-portocaval shunts may result in excessive diversion of the portal flow into the systemic circulation and may lead to the steal phenomenon. Troisi et al. recommended measuring portal flow and calibrating the size of the shunt accordingly [75]. It remains controversial whether these shunts should be closed after graft regeneration occurs and liver function stabilizes [76, 77].

## Pharmacologic manipulation

Pharmacologic manipulation is another method of portal flow modulation. This method is reversible, unlike surgical procedures such as splenectomy or SAL. Propranolol and somatostatin decrease both portal flow and portal pressure reliably and reproducibly in cirrhotic patients [78]; therefore, these drugs are used widely in the treatment of variceal bleeding [79]. A couple of studies reported the effect of somatostatin infusion in an animal model of LT with SFSG [80, 81]. Xu et al. found that somatostatin improved 7-day graft survival by attenuating acute-phase shear stress. Hessheimer et al. revealed that somatostatin reduced portal vein flow and protected sinusoidal endothelial cells, and that somatostatin had a cytoprotective effect on hepatic stellate cells [81]. In a recent randomized trial, Troisi et al. found that somatostatin decreased the hepatic venous portal gradient and preserved arterial flow to the graft [82]. The beneficial effects of the intraportal infusion of prostaglandin E1 (PGE1), a vasodilator with hepatoprotective effects, have been reported [83, 84]. We previously reported that the continuous intraportal infusion of PGE1, nafamostat mesylate, and a thromboxane synthetase inhibitor prevented SFSG syndrome after LDLT by attenuating microcirculatory insufficiency [85]. Moreover, the continuous intraportal infusion of PGE1 was effective in split LT for two adults [86]. Microthrombus in the portal vein, caused by inserting an indwelling catheter when performing continuous intraportal infusion therapy, should be avoided.

In conclusion, portal flow modulation with a prudent combination of shunt control such as ligation or hemi-portocaval shunt, a portal decompression procedure such as simultaneous splenectomy or SAL, and pharmacologic manipulation, is crucial when performing LDLT with an SFSG.

## Conflict of interest

Tomoharu Yoshizumi and Masaki Mori have no conflicts of interest to declare.

## Abbreviations

LDLT: Living donor liver transplantation
SFSG: Small-for-size graft
GRWR: Graft-to-recipient weight ratio
GV: Graft volume
SLV: Standard liver volume
LT: Liver transplantation
PVT: Portal vein thrombosis
SAL: Splenic artery ligation
HVOO: Hepatic venous outflow obstruction
PGE1: Prostaglandin E1
