# Correction to: Portal flow modulation in living donor liver transplantation: review with a focus on splenectomy

**DOI:** 10.1007/s00595-019-01949-9

**Published:** 2020-01-08

**Authors:** Tomoharu Yoshizumi, Masaki Mori

**Affiliations:** Department of Surgery and Science, Graduate School of Medical Sciences, Kyushu University, 3-1-1 Maidashi, Higashi-ku, Fukuoka, 812-8582 Japan

## Correction to: Surgery Today (2020) 50:21–29 https://doi.org/10.1007/s00595-019-01881-y

 The article Portal flow modulation in living donor liver transplantation: review with a focus on splenectomy, written by Tomoharu Yoshizumi and Masaki Mori, was originally published Online First without Open Access. After publication in volume 50, issue 1, page 21–29 the author decided to opt for Open Choice and to make the article an Open Access publication. Therefore, the copyright of the article has been changed to © The Author(s) 2020 and the article is forthwith distributed under the terms of the Creative Commons Attribution 4.0 International License (https://creativecommons.org/licenses/by/4.0/), which permits use, duplication, adaptation, distribution and reproduction in any medium or format, as long as you give appropriate credit to the original author(s) and the source, provide a link to the Creative Commons license, and indicate if changes were made.

The original article has been corrected.

